# Zika Virus: A New Animal Model for an Arbovirus

**DOI:** 10.1371/journal.pntd.0004702

**Published:** 2016-05-05

**Authors:** M. Javad Aman, Fatah Kashanchi

**Affiliations:** 1 Integrated BioTherapeutics, Inc., Gaithersburg, Maryland, United States of America; 2 Laboratory of Molecular Virology, George Mason University, Manassas, Virginia, United States of America; Colorado State University, UNITED STATES

In the latest issue of *PLOS Neglected Tropical Diseases* [1], Dowall and colleagues describe a novel small animal model for Zika virus infection. Along with two other reports in the recent weeks [2,3], this collection of papers marks an important turning point in Zika virus research and enables *in vivo* testing and evaluation of candidate vaccines and therapeutics. The authors show that type-1 interferon receptor deficient mice (A129), in contrast to the parental strain (129Sv/Ev), are susceptible to Zika infection.

Zika virus was first isolated from the serum of a sentinel rhesus monkey in 1947 in Zika forest, Uganda [4]. The second more confirmatory Zika isolates were recovered from a pool of *Aedes (Stegomyia) africanus* (Theobald) mosquitoes from the same region in 1948 [5]. Zika virus (ZIKV) is an arthropod-born virus (arbovirus) belonging to the genus *Flavivirus* and the family of *Flaviviridae*. The genus comprises of some 52 other viral species, including more pathogenic viruses such as dengue, yellow fever, St. Louis encephalitis, tick borne encephalitis, and West Nile Viruses. The virions are small, spherical in shape containing a positive single stranded non-segmented RNA of approximately 11kb. The genome contains 2 flanking non-coding regions (NCR; a 5’ 59 base and a 3’ 39 base) a single open reading frame that codes for capsid (C), precursor membrane (PrM), envelope (E) and seven non-structural proteins formed NS1, 2A, 2B, 3, 4A, 4B, and 5.

The significance of mosquito-borne transmission of Zika virus has been experimentally demonstrated in a study in which *Aedes aegypti* mosquitoes, artificially fed with Zika virus, were able to transmit the virus to mice and monkeys [6]. While transmission of ZIKV from infected female mosquitoes to susceptible vertebrate hosts is common, evidence for non-vector-borne transmission has been shown through the presence of viral RNA in human urine, placental tissue, amniotic fluid, semen, and saliva. These findings represent the additional potential for ZIKV to be transmitted human-to-human [7]. The pathologies associated with Zika virus are generally mild or present with symptoms typical for dengue virus infection such as fever, prostration, general muscle aches, maculopapular rash, and eye pain. The virus in general does not cause hemorrhagic fever or death. A clear Zika epidemic is difficult to establish since the general symptoms resemble dengue and chikungunya, making clinical diagnostics somewhat unreliable. However, several Zika outbreaks have been identified over the past ten years in the Pacific islands and more recently in Brazil which rapidly spread to 25 countries in the region [8]. While ZIKV infection is generally mild or asymptomatic an association between ZIKV and the autoimmune Guillain- Barré syndrome, established in 2013 outbreak in French Polynesia [7] caused concern about serious sequelae in humans. This concern was further aggravated by the observation of 20-fold increase in incidence of congenital microcephaly during the 2015 outbreak in Brazil [8]. The surviving children with microcephaly will potentially have developmental issues and many will need life-long support, thus having a major socio-economic impact. Furthermore, there is a serious concern about potential transmission through sexual contact as well as blood transfusion. Due to the rapid spread of the virus, the potential for human to human transmission, and the serious complications WHO formally declared the current ZIKV epidemic as a public health emergency of international concern. A comprehensive and constantly updated website is available on the Pan American Health Organization (PAHO; http://www.paho.org/hq/).

The manuscript by Dowall et al., utilizes female immuno-deficient mice lacking the IFNα/B receptor (A129) and congenic control mice (129SV/EV) for ZIKV infection. The un-adapted viral inocula used was injected at 10^6^ plaque-forming units (Pfu) in the right and left hind legs, which is reminiscent of the viral dose level of 10^4^–10^6^ per mosquito bite shown for West Nile virus [1]. The readouts included survival, clinical numerical scores at each time point of observations, temperature and weight changes, and detection of RNA at necropsy in blood and tissue. All A129 mice challenged with ZIKV succumbed to infection within 6 days. These mice exhibited weight loss after 3 days, slightly increased temperature by day 4, followed by hypothermia, and detected viral RNA on day 3 and 6 in blood, spleen, liver, ovary, and brain. Viral RNA levels ranging from ~10^7^ in blood and liver to nearly 10^11^ in brain are within the range of viral RNA loads detected in human serum and breast milk (10^6^), semen (10^7^), and urine (10^8^) [7]. All surviving control 129Sv/Ev animals showed low levels of viral RNA in the blood, ovary, and spleen but the virus did not appear to cross the blood brain barrier. In the spleen of susceptible A129 mice they observed poorly defined germinal centers with numerous apoptotic bodies. Importantly, the authors also observed ocular and CNS abnormalities which included nuclear fragmentation in grey and white matter, perivascular cuffing of vessels in meninges, and presence of hyper-eosinophilic cytoplasm among neurons of the hippocampus. Future research should show to what extent these symptoms reflect the CNS abnormalities in humans.

While Dowall et al. [1] utilized the African strain MP1751, Lazear et al. and Rossi et al. [2,3] demonstrated that the A129 model can also recapitulate the disease caused by the Asian strains FSS1325 and H/PF/2013. This is significant since the recent outbreaks were all caused by the Asian lineage of Zika virus.

In many respects, Zika infection may be reminiscent of another significant public health hazard, namely German measles [9]. Rubella virus is the causative agent of Rubella disease or German measles, which infects the fetus at the first trimester resulting in miscarriage or congenital rubella syndrome (CRS) [10]. The virus can be transmitted from person to person via the respiratory route and can cause demyelination in rat brain cells [11]. The syndrome is characterized by pathologies of the eye (microphthalmia, pigmentary retinopathy, and chorioretinitis), heart (peripheral pulmonary artery stenosis, and ventricular septal defects), as well as, brain (microcephaly). More importantly, surviving neonates can face serious developmental disabilities including visual and hearing impairments, and increase in developmental delay exemplified in autism. It remains to be seen if, like CRS, ZIKV infection also induces such a wide range of congenital neurological pathologies. Vaccination against Rubella has largely wiped out these severe consequences, although, there are still outbreaks in regions that do not adhere to a complete vaccine program (i.e. Japan or India). It is hoped that a successful future vaccine for ZIKV will do the same for complications of this newly emerging virus.

Finally, the animal model described in the Dowall and colleagues manuscript [1] can potentially open the door for answering many critical scientific questions for differential tissue diagnostics between various viral strains; specific (viral) or non-specific (host) therapeutics against ZIKV (which may have many similar phenotypes compared to other Flaviviruses); modes of transmission through aerosol, saliva, blood, or sexual contact; determining surrogate markers of infection; long term cell mediated immunity; specific antibodies against key antigens that affect CNS (i.e. similar to Rubella E1 protein binding to myelin oligodendrocyte glycoprotein, MOG); and key issues related to inter individual variabilities in the innate and acquired immune response phenotypes (i.e., PD-1 immune check point) to Zika-containing vaccine. Lessons learned from CRS are likely to be very helpful in design of animal studies aimed at understanding the pathophysiology of Zika virus.

While the A129 model lacks a fully competent immune system, it will likely be suitable for testing therapeutics and some vaccine studies [12]. In this regard, recent demonstration of ZIKV replication in immunocompetent mice treated with neutralizing monoclonal antibodies against IFN may represent an alternative for ZIKV vaccine research [2]. It is further important to develop models that can recapitulate the congenital sequelae of ZIKV infection. Such models have to allow for non-lethal or asymptomatic infection of pregnant mice. In all, the latest arboviral spread as seen by ZIKV may be better managed and cured using such animal models to achieve a fast and reliable solution compared to the present mainstay of management which includes bed rest and supportive care.
